# Indocyanine green fluorescence in second-look surgery for necrotizing enterocolitis: enhancing the surgeon's perception

**DOI:** 10.3389/fped.2025.1641794

**Published:** 2025-09-29

**Authors:** Antonia Maximina Pfennigs, Manuel Besendörfer, Sonja Diez, Bertram Reingruber

**Affiliations:** ^1^ Clinic for Pediatric Surgery and Traumatology, Florence Nightingale Hospital, Düsseldorf, Germany; ^2^ Department of Pediatric Surgery, University Hospital Erlangen, Friedrich-Alexander-Universität Erlangen-Nürnberg, Erlangen, Germany

**Keywords:** necrotizing enterocolitis, indocyanine green, fluorescence, blood perfusion, short bowel syndrome

## Abstract

**Background:**

A perfusion mismatch in the premature gut is the key component in the development of necrotizing enterocolitis (NEC). Resulting necroses need to be surgically excised, while intestinal salvage is crucial to the survival and rehabilitation of affected preterm neonates. Until now, resection margins have been based on standard visual inspection and surgical experience due to the lack of objective criteria for bowel viability. We hypothesize that by evaluating the vitality and perfusion during NEC surgery, necrosis margins can reliably be defined by indocyanine green (ICG) fluorescent imaging, a real-time visualization method, which has already been implemented safely in pediatric surgery for other indications.

**Materials and methods:**

In a prospective study at our Level 1 Perinatal Center, patients were recruited after primary emergency surgery confirming NEC. Due to the acute phase of the inflammatory process, the extent and dynamics cannot be clearly defined at this point. Informed consent of the parents for the second-look surgery included ICG fluorescent imaging (0.04–0.7 mg/kg body weight), which was applied after completely exposing the intestinal bundle to visualize blood perfusion intraoperatively. ICG perfusion of intestinal tissue was visualized by a near-infrared camera and was compared with bowel vitality as judged conventionally by an experienced surgeon. Based on the findings, further treatment was specified. We correlated our surgical findings with subsequent histopathology.

**Results:**

Six patients treated at our perinatal center met the inclusion criteria. In four patients, ICG-negative, non-vital gut areas were detected and resected with narrow margins of <1 mm. Histology and the further medical course proved to be consistent with these intraoperative results. In two patients, the clinical appearance of complete necrosis of the small intestines was confirmed by ICG fluorescence, supporting the decision to provide palliative treatment. In two out of six patients, clinical judgment and real-time ICG fluorescence were contradictory. Here, histopathology confirmed complete necrosis of the bowel in full accordance with ICG.

**Conclusion:**

Our prospective cohort study gave evidence for ICG fluorescence to be useful and reliable in objectifying blood perfusion and intestinal vitality during NEC surgery, adding objectiveness to the surgeon's personal experience.

## 1 Introduction

Conditions consistent with necrotizing enterocolitis (NEC) were described two centuries ago by Charles Billard (1). Although there have been improvements in the care of neonates, especially those with very low birth weight (VLBW) or extremely low birth weight (ELBW), approximately 7% of VLBW infants in neonatal intensive care units (NICU) develop NEC (2–4).

Bell et al. (5) published a classification system for the diagnosis and staging of NEC in 1978, which was modified a few years later. Proven NEC at Bell Stage III, also referred to as advanced or surgical NEC, requires surgical intervention. Clinical trials showed no significant difference in the outcomes of interventional placement of an abdominal drainage to open surgical exploration (6). By the nature of the procedure, laparotomy provides a good overview of the spreading pattern and the severity of the disease. In any case, surgery should not take more than 45 min to minimize stress on the frail organism (7). Several revisions are usually necessary (8–11). Essential to all surgical procedures is the preservation of the maximum length of bowel, while avoiding intestinal spillage and sparing the organism from inflammation and the metabolic burden of necrosis (12–15). Limiting necrosectomy and maximizing bowel preservation are crucial, and yet it is challenging to avoid early complications such as anastomotic leakage and late complications such as intestinal failure and short bowel syndrome (SBS).

As neonatal care has improved, the incidence of NEC has declined. Case numbers dropped by nearly 50% over the last two decades in Germany (16), and there was a corresponding drop in the surgical intervention rate. Consequently, surgical training and the multiplication of surgical experience in NEC surgery have become challenging. With less practice, the surgeon's ability to assess gut recovery may decline, potentially provoking multiple revision laparotomies. New methods for intraoperative judgment of intestinal vitality, including the pattern and the extent of visceral blood perfusion, may provide enough safety to refrain from multiple revisions. This technique should be sensitive, specific, reliable, and safe, especially in the early phase of the disease, as a conventional and clearly visible clinical demarcation of necrotic bowel tissue can take several days to develop and might otherwise increase the number of revision laparotomies.

A method to visualize intestinal perfusion via near-infrared fluorescence optical imaging (FOI) is indocyanine green (ICG) optical imaging. Originally developed for near-infrared photography, ICG was approved for clinical use in 1956. Binding with blood lipoproteins, ICG shows little tissue leakage, making it ideal to visualize tissue blood perfusion. When illuminated at 750–800 nm wavelength, emissions can be observed at a wavelength shortly over 800 nm with a near-infrared camera (17). It has found multiple applications over the last few decades, which led to great improvements in diagnostic methods and surgical outcomes in a variety of applications where perfusion is of key importance, such as anastomosis, tumor margins, chronic inflammation, or visualization of blood circulation patterns (18, 19). ICG is a non-toxic, non-ionic molecule, and its adverse effects, such as allergic flush or discoloration of the skin, range between 0.15% and 0.05% with no deaths reported so far (17, 20). Recent publications have shown that ICG has already found its way into pediatric surgical procedures, where it, for example, can be used to map the thoracic duct during esophageal atresia procedures and to prevent chylothorax or to visualize the perfusion during reconstructive surgery of anorectal malformations or during a Palomo procedure in pediatric urology (21–23). A pilot study using ICG as an additive during the Kasai procedure in neonates with biliary atresia showed an improved postoperative normalization of bilirubin levels (24, 25). Furthermore, in a prospective mixed clinical trial, ICG proved to be a safe and feasible tool for visualizing blood perfusion during pediatric intestinal resections (26).

Piglet models have shown promising advantages and high practicability in assessing intestinal perfusion in induced ischemia and inflammatory processes of the small intestine (27). ICG has been shown to be beneficial in evaluating intestinal perfusion in a piglet model of induced NEC (28). We therefore suggest introducing this well-established method into perinatal surgery and specifically NEC surgery, thereby implementing a tool that will objectify intraoperative and real-time judgment of intestinal viability and perfusion in cases of NEC.

## 2 Materials and methods

### 2.1 Patient population

**Figure 1:**
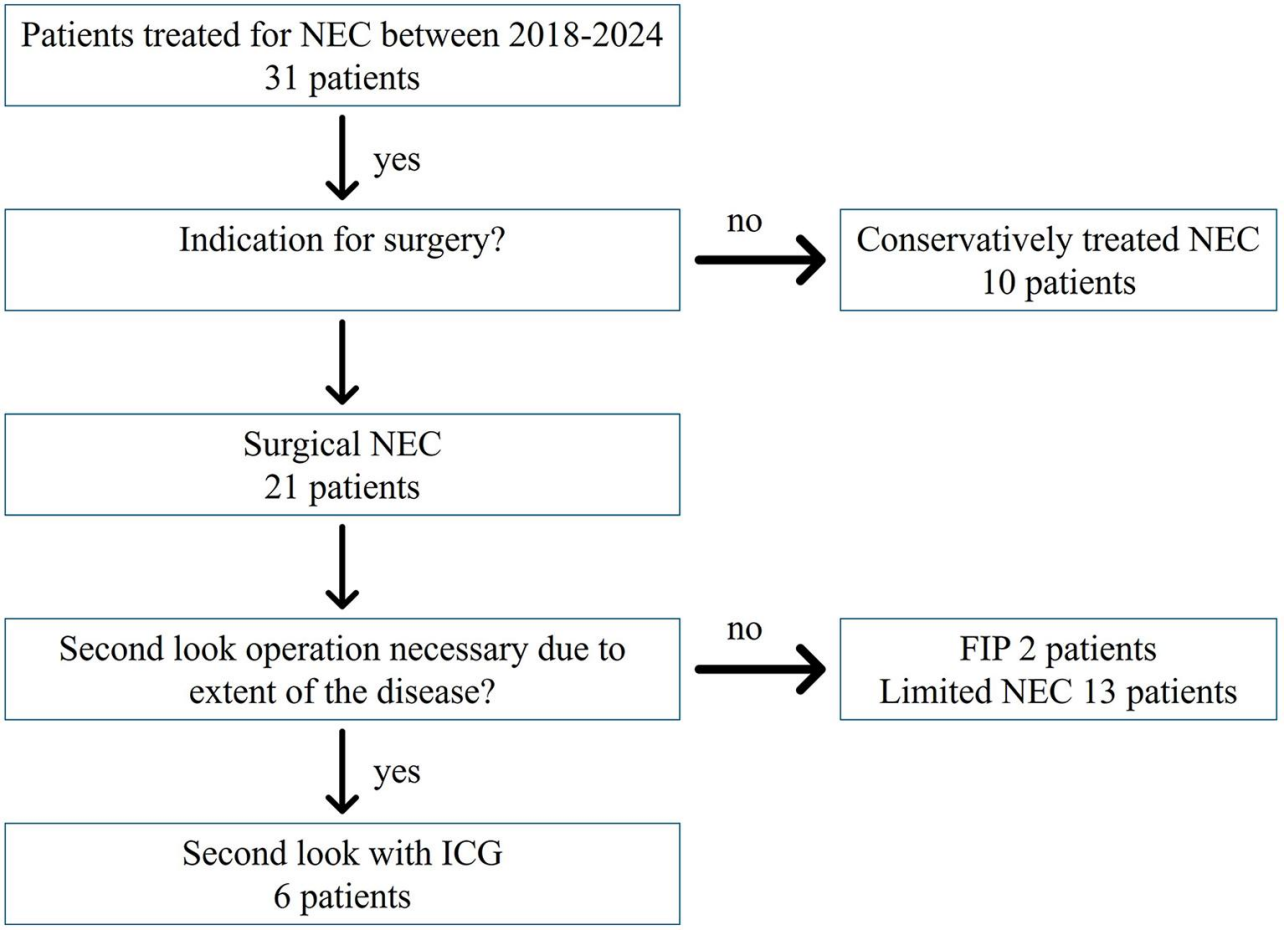
With a total cohort of 31 patients treated for NEC at our hospital, 10 patients were managed conservatively. Out of 21 patients with surgical intervention, 2 had a FIP, and 13 had only limited NEC. Six patients underwent a second-look operation with ICG and were analyzed for this study.

The study's cohort was generated prospectively, including patients treated from 2018 to 2024 in the NICU department of the Florence Nightingale Hospital, Düsseldorf, Germany. The inclusion criteria included NEC Stage Bell III with indication for surgical treatment in the neonatal period and extensive bowel inflammation over a major portion of the intestines, necessitating a second-look procedure. Furthermore, imminent short gut syndrome or complete bowel ischemia were used as criteria for inclusion, whereas patients with a substantial length of healthy gut were excluded. Patients with focal intestinal perforation (FIP), meconium ileus, and limited, uni-segmental or focal NEC were excluded, as they would not benefit from a second-look operation (Figure 1). Informed consent was obtained for all participants from their next of kin.

### 2.2 Clinical findings, diagnostic imaging, and indication for surgery

Patients received enteral feeding either via nasogastric tube or per mouth with breast milk or, if not available, preterm infant formula and NEC prevention with probiotics according to the standard in our German Level 1 NICU (corresponding levels: UK: 3; USA: 4) (29). Diagnostic imaging upon suspected NEC included bedside ultrasound with findings of portal venous gas and bedside x-rays to look for pneumoperitoneum. In case of NEC diagnosis, conservative treatment was initiated, including antibiotic treatment and fasting. To monitor disease progression, blood tests included leucocyte count, C-reactive protein, and IL6. There was an interdisciplinary discussion about the indication for surgery, considering the signs of pneumoperitoneum and clinical deterioration despite fully escalated conservative management.

Primary exploratory laparotomy was performed bedside in the NICU by an experienced pediatric surgeon, and unmistakably necrotic and disintegrated bowel was removed. Any bowel segments still showing intact wall structures were preserved, even if appearing non-vital, and a second-look procedure was scheduled 2–4 days later. A bowel ostomy was created at the most proximal point of the disease. If the extent of the disease in the first exploratory surgery was of critical extent, suggesting that ICG visualization could be beneficial to improve the identification of resection margins in the second-look operation, the second-look procedure was planned with ICG. In between the procedures, the patients received parenteral nutrition and empirical antibiotic treatment with vancomycin and meropenem, which was adapted based on the subsequent pattern of antibiotic sensitivity further on. Patients were continuously monitored in the NICU, thereby optimizing blood pressure and oxygenation, while remaining intubated and mechanically ventilated.

### 2.3 Application of ICG

**Figure 2:**
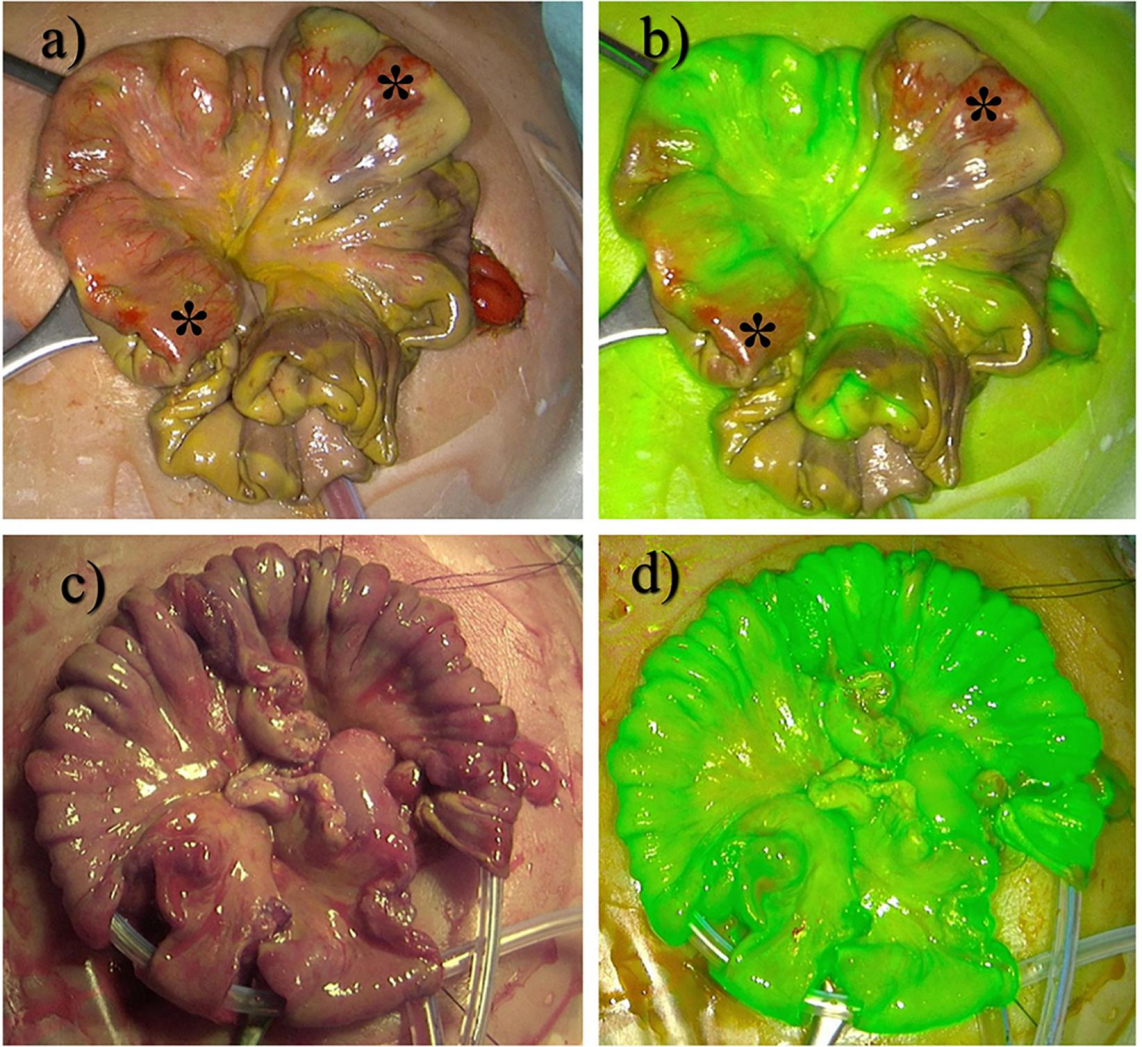
**(a)** Intraoperative site of a patient with NEC Stage Bell III. **(b)** ICG visualization of **(a)**. **(c)** Successful resection of necrotic areas. **(d)** ICG fluorescence control after resection. *indicates non-perfused areas still looking vital.

ICG visualization was performed during the scheduled second-look surgery either in the NICU or operating room, depending on the patient's hemodynamic stability. Upon second-look laparotomy and after adhesiolysis of the intestines, 0.04–0.7 mg/kg Verdye® (Diagnostic Green Ltd., Athlone, Westmeath, Ireland) according to the manufacturer's recommendations was intravenously applied unfiltered by the anesthesiologist once the bowel was fully exposed. As a useful monitoring device to track the onset of ICG flow, we used the oxygen saturation sensor, which monitors ventilation and oxygenation by the anesthesiologist as part of the regular anesthesia setup. The flow and spreading of ICG were monitored through subtle changes within a few seconds in the sensor, as this also operates within the near-infrared spectrum. The effect of the ICG on the sensor was observed as a drop in the saturation of <15%, only lasting a few seconds, while the ICG fluorescence persisted. Visualization of intestinal perfusion was achieved using either the mobile Novadaq® Pinpoint Endoscopic Fluorescence imaging system or the Storz Image1 4K endoscopic tower, immediately after application of ICG. With the assistant holding the camera device at a 90° angle, approximately 30 cm above the exposed bowel, the intestine was systematically inspected by the senior surgeon. The direct clinical appearance in the operating field was compared with the blood perfusion as visualized by ICG imaging. The colon and stomach served as reliable positive controls for a solid ICG signal. Any fluorescence signal similar to the stomach and colon was regarded as a positive signal; a faint or non-existent signal was regarded as ICG negative. All non-perfused segments of bowel were resected precisely at their demarcation. Finally, ICG imaging was conducted to confirm perfusion of the remaining bowel. Surgical sites were documented by digital photography (Figure 2).

### 2.4 Statistical analyses

Data were recorded as quantitative variables or categorical factors. For quantitative or ordinally scaled data, the median value together with the minimum and maximum values were given. For qualitative factors, absolute and relative frequencies were assessed. Imaging ICG diagnostics were compared with macroscopic intra-abdominal findings during the surgical procedures.

## 3 Results

### 3.1 Demographic data

Between 2018 and 2024, a total of 31 patients were treated for NEC in our hospital. In 21 patients (68%), the indication for surgery was Bell Stage III. Of the 21 patients, 2 (10%) whose intraoperative findings showed a FIP and 13 (62%) with focal or limited NEC were excluded from the case series. In 6 (29%) out of 21 patients, intraoperative findings showed extensive disease, meeting our criteria for ICG imaging. Demographic data are summarized in Table 1. Patients included were born between 24 and 27 weeks of gestational age, with a median age at surgery of 24 days (range 4–44 days). Birth weight ranged between 400 and 1,030 g with a median of 1,700 g.

**Table 1:** Demographic data of the participants.

ID	Sex	Gestational age (weeks + days)	Birth weight (g)	Left-to-right shunt	Probiotics prior to NEC	Feeding prior to NEC	Preoperative free air	Age at first surgery (days)	Weight at surgery (g)	Total number of surgeries for NEC
1	f	24 + 2	490	PDA	Yes	Human milk	Yes	35	880	4
2	f	26 + 6	520	PDA	Yes	Formula	Yes	14	600	2
3	f	26 + 1	400	PDA	Yes	Enteral tolerance too bad for feeding	No	10	438	2
4	f	24 + 1	620	None	Yes	Human milk/formula	No	34	970	3
5	f	27 + 0	1,030	PDA	Yes	Formula	No	44	1,500	3
6	m	26 + 2	690	PDA + PFO	Yes	Human milk	No	8	700	3

f, female; m, male; PDA, patent ductus arteriosus; PFO, patent foramen ovale.

### 3.2 Surgical management

To increase reproducibility, the comparison between macroscopic findings and ICG was carried out by the same senior surgeon. First-look surgery of our study cohort without ICG was 75 min (range 36–144 min), median operation time in second-look surgery including ICG was 11% higher at 83 min (range 27–228 min).

In four out of six patients in the study group, clinical judgment of critical bowel lesions was consistent with ICG imaging, leading to resection in one session and restoring continuity by end-to-end anastomoses. In two of these patients, ICG imaging showed no perfusion, whereas the intestine appeared healthy on standard visual inspection. These areas were inspected thoroughly by the operating senior surgeon, which were subsequently proven non-vital by carefully controlled incision and absence of bleeding and therefore dissected. Histopathology later confirmed these findings. A reason for this mismatch of the surgeon's perception and the ICG signal could not be identified, as there were no differences regarding disease severity or medical handling, including a constant team of medical personnel. A diverting ostomy was created at the most proximal defect to allow for early enteral feeding while the multiple anastomoses of the distal, aboral intestine were allowed to heal. Antibiotics were continued and, if necessary, adapted to microbiological findings.

**Figure 3:**
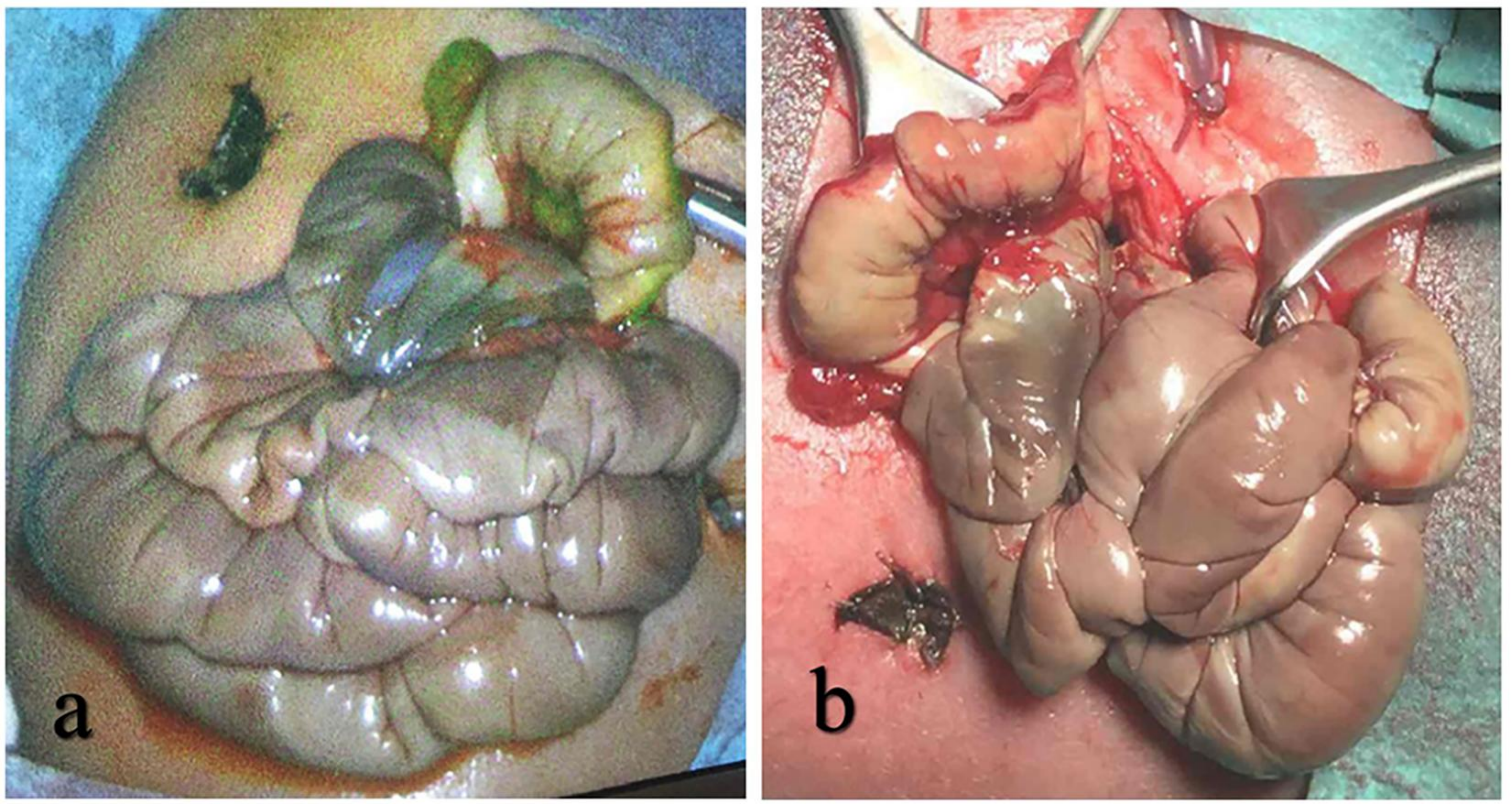
(**a**) ICG fluorescence shows no signal of blood perfusion. (**b**) Examination of the gut reveals non-vital and necrotic tissue.

In the other two patients, complete ischemia of the entire small intestine was already suspected at the primary operation. A diverting proximal jejunostomy was performed. By the time of the scheduled second-look operation, the whole small intestine was thoroughly examined macroscopically and via ICG for any signs of substantial blood perfusion and was judged completely ischemic on macroscopic examination, as well as by ICG imaging (Figure 3). The presumed occlusion of the superior mesenteric artery resulting in complete bowel necrosis was subsequently confirmed by histopathology. Surgical findings are summarized in Table 2.

**Table 2:** Surgical data and outcome of participants.

ID	Remaining small bowel length, first look (cm)	Interval first- and second-look surgery	Remaining small bowel length, second look (cm)	Dose of ICG	Number of anastomoses/insufficiencies	Conflict of macroscopic finding vs. ICG?	SBS?	Follow-up time (days or months)	Outcome (death/SBS/total enteral nutrition)	Conflict between ICG and histopathological findings
1	40	4	20	0.15 mg	8/none	Yes	Yes	45 months	Enteral nutrition	No
2	30	2	None	0.12 mg	None/none	No	Yes	16 days	Death	No
3	10	2	7	0.1 mg	None/none	No	Yes	13 days	Death	No
4	nb	3	20	0.2 mg	1/none	Yes	No	8 months	Enteral nutrition	No
5	nb	3	>20	0.75 mg	3/none	No	Yes	22 months	Enteral nutrition	No
6	15	4	12	0.15 mg	3/none	No	Yes	11 months	SBS in weaning	No

SBS, short bowel syndrome; nb, not described.

### 3.3 Postoperative management and follow-up

Histopathological examination of the resected specimens confirmed complete necrosis with barely recognizable wall structures or full-thickness bowel necrosis and extensive pneumatosis intestinalis, notably so in all areas where a negative ICG signal stood in contrast to conventional macroscopic findings.

Oral feeding started only a few days after surgery. Once the critical phase was managed, radiologic contrast imaging of the efferent bowel loop was carried out to confirm bowel sufficiency. Antibiotic treatment was terminated once blood parameters had normalized. The defunctioning stoma was reversed in a limited local procedure after 8–21 weeks.

Two patients died within 2 weeks of second-look surgery under palliative care following the occlusion of the superior mesenteric artery. Follow-up of the four remaining patients was conducted at a median interval of 16.5 months (range 8–45 months). SBS persisted for 7–9 months postoperatively in all four patients. At the final follow-up, the weaning process of parenteral nutrition was successful in 75% (*n* = 3), with one patient in need of parenteral nutrition for a few hours during the night. In summary, in the six patients evaluated with ICG during second-look surgery, the operating surgeon's perception was supported in four out of six cases. In two out of six cases, ICG proved to be superior to the surgeon's assessment of the gut, where a negative ICG signal was confirmed as a complete necrosis afterwards by histopathology. We monitored our patients closely for potential side effects such as signs of an allergic reaction due to the dye's iodine base or persistent discoloration of any tissue. No side effects of ICG described in the literature, such as allergic reaction, flush, or persistent discoloration of the skin, were detected during the course of our study.

## 4 Discussion

We present a prospective study of intravenous application of ICG in six preterm infants with NEC aiming to improve assessment of gut perfusion during second-look surgery. In our study, ICG appeared to be superior in terms of indicating blood perfusion and tissue vitality, even to the judgment of an experienced senior pediatric surgeon. Only a few comparable studies exist, none of which investigated the safety and benefit of ICG for this indication and in this population and age group. Previous studies were able to demonstrate that the use of ICG in neonates is safe for multiple indications, including surgery in biliary atresia and esophageal atresia (17). A study conducted in Canada investigated the safety and feasibility of ICG imaging in pediatric bowel resections in general, including, but not particularly for, NEC. A substantial ICG signal could be observed even during emergency surgeries and NEC surgery in premature infants. While the general safety and feasibility of the method could be confirmed, a higher mismatch rate of 62% compared with our mismatch rate of 33% between the surgeon's perception and ICG fluorescence was described (26).

Even though ICG has been applied for various surgical indications within adult surgery for more than half a century, its implementation in pediatric surgery started less than half a decade ago. It has been approved for intravenous use in adults, adolescents, and children (21–23). We hypothesize its feasibility in a neonatal population with NEC based on the following aspects:

1. Whenever surgery is indicated in NEC, its primary aim should be to preserve as much bowel as possible while clearing the abdomen and the organism from the metabolic load of toxic bowel necrosis. SBS is a severe, life-altering, and determining condition, which must be avoided by any means. Any additional diagnostic measure to visualize intestinal perfusion might help improve the outcome of this population and prevent an excessive bowel resection. It can be discussed whether ICG might already add benefit during the primary exploratory surgery. However, the primary surgery in NEC is characterized as a short damage control procedure within an ongoing inflammatory process, aiming at intestinal rescue by diversion ostomy as a gentle procedure with the shortest possible operating time to protect the frail organism. Second-look surgery, as a scheduled surgery after several days of stabilization, presents an opportunity to take reconstructive steps. Improved identification of the resection margins of vital bowel by visualizing blood perfusion via ICG fluorescence can help avoid excessive resection and promote bowel reconstruction by avoiding multiple revision operations. This aligns with the observations previously described in the literature, where areas with mixed ICG signal during NEC surgery showed a subsequent stenosis on a revision later on (26).
2. As understanding, prophylaxis, and conservative treatment of NEC continue to improve, the incidence of advanced NEC in Germany declines, as well as the number and proportion of surgical interventions and therefore surgical experience for this condition in general. Therapeutic success depends on experience and diagnostic precision. Even with long surgical experience, the real-time judgment of intestinal perfusion can be challenging and, if indeterminate, might demand several revision laparotomies or risk an extensive loss of intestinal length. Intraoperative fluorescent imaging with ICG supports the discrimination of intestinal tissue worth preserving via real-time blood perfusion in a non-invasive, radiation-free manner to increase diagnostic precision safety and thereby assist clinical judgment in case of prognostic uncertainty. In two cases in our study, ICG proved to be superior even to the experienced surgeon's eye, facilitating an easier and quicker removal of non-vital gut sections. Histopathology showed a full necrosis in these cases. However, these cases were neither more severe nor were the blood circulation exceedingly impaired compared with the other cases. ICG might therefore have shown a perfusion mismatch before the full necrotic demarcation took place.
3. Centralization in healthcare is often used to aggregate surgical experience when facing small operative numbers in elective procedures. However, any centralization of NEC treatment in specialized, high-volume hospitals must be weighed against studies that have shown that emergency transport prior to surgery might not only cause time delay but also generate additional complications and increase mortality of these frail neonates (30–32).

Having been used for retinal screening and measuring cardiac output since the 1960s, ICG has more recently found its way into several segments of surgical and interventional medicine (33). It proved useful to detect sentinel lymph nodes in breast cancer, to clarify anatomic structures in hepatobiliary surgery, and to increase the safety of anastomoses in colorectal surgery. Lately, it has also been successfully implemented in emergency surgery, and a variety of handheld or endoscopic camera-bound visualizing devices are on the market (34–39). Recently, it has found its way into pediatric surgery and urology, with so far limited experience in neonates and preterm infants (21, 23). A potentially beneficial role in the context of NEC surgery has been shown by Knudsen et al. (28) in a piglet model. Based on the experience of over sixty years, ICG acts with only a few side effects, such as subcutaneous retention and rare allergic reactions, and appears safe to use in the latest pediatric analyses (26, 40, 41). However, larger population studies are also needed to address possible side effects. In our study, ICG proved to be superior to the surgeon's assessment alone, as some portions of bowel, conventionally judged vital, showed no ICG signal. All areas demonstrated no bleeding upon incision and proved to be necrotic in histopathological examination. However, the surgeon's assessment should remain cautious as reduced perfusion rates in hemodynamically unstable patients might have an influence on ICG distribution and therefore its value. Further studies are needed to evaluate this important aspect.

Surgery for NEC can be challenging even for the experienced surgeon, and the outcome is often dependent on subjective variables and intraoperative decisions. Therefore, a reliable, efficient, and widely available diagnostic tool such as ICG may not only compensate for decreasing surgical exposure to this condition but may also decisively contribute to improving intraoperative decision making, standardization, and possibly even case discussion via telemedicine. Prospectively, ICG visualization could add safety in cases with limited NEC, which could be suitable for primary anastomosis. This might be especially interesting for higher volume centers, in which ICG might reduce a dependence on subjective interpretation of gut vitality. In addition, in the case of defining fatal intestinal ischemia, the extent of the disease can be visualized and demonstrated objectively and earlier in its course. In these cases, therapeutic limits can be discussed with the parents under ethical criteria, avoiding prolonged periods of uncertainty.

This study has several limitations. Based on the low incidence of NEC, the study population is small. Although we included the study's participants prospectively, indications for ICG might be biased in this heterogeneous population, including a large age span at primary operation, cardiac abnormalities, and differences in feeding. A control group without ICG would have been helpful to show whether the application of the ICG procedure time or shortens the operating time by improving efficiency. Other aspects of interest would be a comparison of the potential benefits of time to oral feeding in the ICG group, length of hospital stay, and length of dependence on parenteral nutrition. A historical matched pair analysis would be useful; however, due to the individuality of each case, comparability might be limited. In addition, ICG was solely administered during second-look surgery in our study population. Therefore, the patients had already reached stability in blood circulatory support. Additional exploratory research is needed to ascertain whether the ICG signal is reliable when used in a hemodynamically unstable patient. In general, ICG visualization has proven to be safe and feasible in NEC surgery in our institution, and we will continue to recruit patients and to increase data on the benefit of this new indication. Multicentric studies would also be desirable.

## 5 Summary

ICG fluorescence imaging is a widely available and safe diagnostic tool. With its reliability in visualizing real-time intestinal perfusion, ICG can be used to significantly improve the judgment of gut viability during exploratory laparotomy, as could be demonstrated in this present prospective study. This allows for an early and precise definition of the resection margins of necrotic bowel, regardless of patient specificities or the individual experience of the performing surgeon. Hereby, the metabolic burden of gut necrosis can be eliminated early, and the number of surgical interventions can be minimized without sacrificing bowel length preemptively or due to the limits of conventional discrimination in surgical perception. ICG can support the prognosis of intestinal restitution and, if unfavorable, support a decision for palliative treatment to avoid unnecessary suffering. In the future, a multicenter study comparing outcomes considering overall survival, enteric independence, and time to recovery between ICG-guided resection and conventional procedures could further illuminate the benefit of ICG in NEC surgery while also evaluating possible adverse effects of ICG administration.

## Data Availability

The original contributions presented in the study are included in the article/Supplementary Material; further inquiries can be directed to the corresponding author.

## Author's note

The present work was performed in fulfillment of the requirements for obtaining the degree “Dr. med.” of Antonia Maximina Pfennigs.
